# Engineering clinically relevant volumes of vascularized bone

**DOI:** 10.1111/jcmm.12569

**Published:** 2015-04-15

**Authors:** Brianna M Roux, Ming-Huei Cheng, Eric M Brey

**Affiliations:** a Department of Biomedical Engineering, Illinois Institute of TechnologyChicago, IL, USA; b Research Service, Edward Hines Jr. V.A. HospitalHines, IL, USA; c Department of Plastic and Reconstructive Surgery, Chang Gung Memorial Hospital, College of Medicine, Chang Gung UniversityTaoyuan, Taiwan; d Center for Tissue Engineering, Chang Gung Memorial HospitalTaoyuan, Taiwan

**Keywords:** vascularization, bone tissue engineering, clinical applications

## Abstract

Vascularization remains one of the most important challenges that must be overcome for tissue engineering to be consistently implemented for reconstruction of large volume bone defects. An extensive vascular network is needed for transport of nutrients, waste and progenitor cells required for remodelling and repair. A variety of tissue engineering strategies have been investigated in an attempt to vascularize tissues, including those applying cells, soluble factor delivery strategies, novel design and optimization of bio-active materials, vascular assembly pre-implantation and surgical techniques. However, many of these strategies face substantial barriers that must be overcome prior to their ultimate translation into clinical application. In this review recent progress in engineering vascularized bone will be presented with an emphasis on clinical feasibility.

- Introduction
  Vascularization and bone formation
  Cellular crosstalk
  Design criteria for clinical success
- Cell based strategies
  Cells and scaffolds
  Cells and soluble factors
  Bioreactors
- Cell-free strategies
  Growth factor delivery
  Scaffold design
- Surgical approaches
- Conclusion

## Introduction

Large volume bony defects resulting from trauma, congenital defects or cancer remain a significant challenge for reconstructive surgeons. Autologous tissue transfer is the standard treatment for such defects, but is hindered by donor site morbidity, risk of infection, poor cosmetic and functional outcome, and reduced graft integrity. Allografts are an insufficient solution due to immune response and a lack of sufficient donor tissues. Synthetic materials suffer from erosion, infection and poor outcome. The ability to engineer vascularized bone graft implants with patient-specific geometries has the potential to be an alternative source for tissues used in reconstruction.

### Vascularization and bone formation

**Figure 1:**
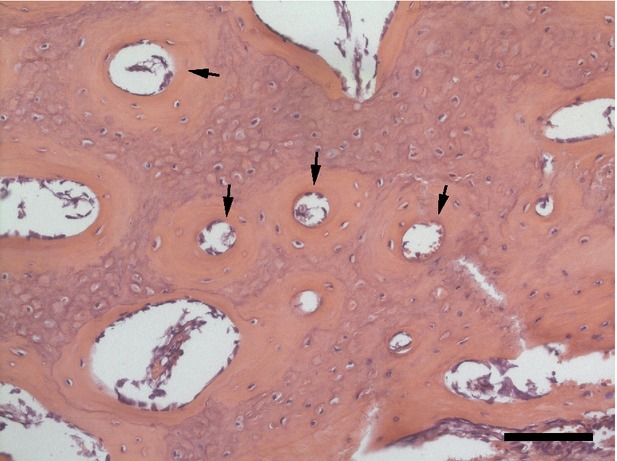
Section of porcine rib stained with haematoxylin and eosin demonstrating the microstructure of bone. Arrows denote Haversian canals. Scale bar represents 100 μm.

Vascular networks are vital to the development, healing and function of bone. The vasculature supplies oxygen and nutrients to the tissue and is a source of osteoprogenitor cells necessary for healing or regeneration in response to local injury. Long bones have a complex hierarchal vascular structure consisting of diaphyseal, metaphyseal, epiphyseal and periosteal arteries. Due to their high metabolic need, osteocytes are typically within 100 μm of a blood vessel 1. Capillaries within Haversian and Volkmann canals supply the osteons (Fig.1), and vasculature in the surrounding periosteum supplies nutrients, oxygen and osteoblast progenitor cells to superficial regions 2,3.

Vascularization and bone formation are highly linked. It is widely agreed that vascularization occurs prior to osteogenesis during both embryonic development and healing of adult bone. During foetal development, mesenchymal precursors in the embryonic limb bud differentiate into chondrocytes, assemble into an avascular cartilaginous bone template and then secrete extracellular matrix and the angiogenic protein VEGF 4. VEGF initiates angiogenesis from nearby vessels, creating a vascular network within the matrix which allows for osteoprogenitor cell migration, differentiation and subsequent bone formation 4. In adult bones, a fibrin clot forms within a damaged region following an injury. The clot serves as a provisional matrix for invasion of vascular networks. This granulation tissue is first replaced by fibrocartilage tissue that is remodelled as bone develops. The extent of bone formation is reduced if the vascularization process is interrupted 5,6.

The vasculature regulates bone formation in a variety of ways. It serves as a source of oxygen, nutrients and progenitor cells but also regulates bone behaviour through direct interactions between endothelial cells (ECs) and bone cells. Endothelial cells induce differentiation of osteoprogenitor cells 7 and enhance osteoblastic gene expression independent of perfusion 8. This is a reciprocal relationship, as osteoblasts stimulate tube-like structure formation of ECs *in vitro* 8 and induce angiogenesis *in vivo* 9, resulting, in part, from secretion of VEGF 10. Mesenchymal stem cells (MSCs), which reside in bone marrow, interact with ECs 11 and promote angiogenesis 12. Mesenchymal stem cells and bone marrow endothelial progenitor cells (EPCs) in co-culture results in increased alkaline phosphatase (ALP) activity, expression of angiogenic and bone markers, and tubulogenesis in comparison to monoculture of either cell type 13. Interactions between vascular and bone cells are vital to the development, function and stabilization of bone.

### Cellular crosstalk

A complex network of communication occurs between ECs and osteoblasts, osteoclasts and osteoprogenitor cells. One of the primary mechanisms by which this communication occurs is through the secretion of soluble factors. Mesenchymal stem cells secrete a number of soluble factors that can influence nearby ECs, including VEGF, angiopoietin-1 (Ang-1), basic fibroblast growth factor, platelet-derived growth factor (PDGF) and insulin-like growth factor-1 (IGF-1) 11,14. VEGF acts on ECs to induce angiogenesis and also binds to VEGF receptors on osteoblasts to induce bone formation 15,16 and stimulate bone repair 9. VEGF receptor-2 (VEGFR2) is expressed in bone tissue, and its activation by VEGF is required for osteoblastic cell proliferation, differentiation and survival 17,18. Bone morphogenetic proteins (BMPs) promote angiogenesis by inducing osteoblastic secretion of VEGF 19, and also play a major role in bone formation and osteoblast differentiation 20,21. Bone morphogenetic protein-2 is involved in the crosstalk between EPCs and MSCs, as it is secreted by MSCs and induces chemotaxis of EPCs 22. Cellular production of osteogenic and angiogenic factors plays a key role in intercellular communication within bone tissue.

Gap junctions allow for direct cytoplasmic connections between two cells and are essential for cellular communication within bone. Connexin43 (Cx43) is the most abundant gap junction protein in bone tissue and plays a critical role in its development and maintenance. Deficiency in Cx43 results in general osteoblast dysfunction and delayed ossification 23. Cx43 is also present in the endothelium of stable microvasculature and a reduction in Cx43 may reduce the angiogenic potential of EPCs 24. Due to the common presence of Cx43 in vasculature and bone, it is widely hypothesized that ECs and osteoblasts communicate through this connection. Human osteoblasts and dermal microvascular ECs can couple through Cx43 25, and the Cx43 coupling of human umbilical vein ECs (HUVECs) and bone marrow stromal cells can regulate osteoblastic gene expression and differentiation 26. Cx43 not only serves to passively bind molecules, but also actively participates in cell signalling process by recruiting signalling factors to influence which signals are transmitted 27. Cell communication *via* Cx43 is essential for the maintenance and function of bone tissue.

### Design criteria for clinical success

Bone regeneration continues to be one of the most active areas of tissue engineering research. It is well-established that vascularization is critical to the field and there are a number of excellent reviews that discuss strategies for engineering vascularized bone 1,5,28–32. However, these reviews primarily focus on developments in basic research with limited discussion of the translational nature of the work. The focus of this review is on the potential for clinical application of tissue engineering strategies under development. The clinical relevance of a particular tissue-engineered bone strategy depends on several factors, including size and volume of the defect/scaffold, mechanical strength, availability of cells, surgical practicality and cost-effectiveness.

In most cases, one primary role of the skeletal system is mechanical support. The strength of any bone tissue implant is fundamental to maintaining appropriate function. The mechanical properties vary significantly within a given bone 33 and between types of bone. The elastic moduli of native trabecular and cortical bone are approximately 10–15 and 18–20 GPa, respectively 34. Craniofacial bone has a mechanical strength ∽2 orders of magnitude lower than long bones. The mandibular condyle has an elastic modulus of approximately 120–440 MPa depending on orientation 35. When an implant has a tensile strength far greater than native bone, stress shielding can occur and result in resorption of surrounding bone due to underutilization 36. In tissue engineering, it is more common that the strength is lower relative to native bone. The polymer scaffolds used are either unable to achieve the appropriate strength or the mechanical properties decrease rapidly after implantation due to degradation. This results in a structurally weak defect prone to failure. Strategies have been proposed in which an implanted engineered bone is combined with a transient support structure that allows for mechanical development *in situ*. The support structure would then be removed once the implanted bone developed sufficient strength.

Engineering bone of sufficient volume to treat large defects commonly encountered in the clinic is one of the most significant barriers to application. Critical-sized defects in simple fractures in humans often result in a 2–3 cm gap 30, and defects resulting from trauma or tumour resection can be much larger. Tissue engineering strategies are commonly evaluated in much smaller volumes than what is required for reconstruction of large clinical defects. For these approaches to be clinically applicable, they will need to be successful when scaled up. The 3D shape of the defect is also of critical importance. Irregular or complex shapes are difficult to match and poor graft fit can lead to non-union with surrounding bone.

The large majority of tissue engineering strategies apply cells to scaffolds to enhance tissue formation. For these techniques to be realized clinically, cell sources would need to be readily available. When evaluating vascularization of tissues, many studies use ‘model’ cell types, such as HUVECs which may form extensive vascular networks *in vitro* and *in vivo* 37,38. However, these cells are not available in the potential patient population. Results discovered with similar ‘model’ cells or cell lines need to be confirmed with autologous cell sources such as EPCs or MSCs, which can be isolated from adults. Mesenchymal stem cells are primarily extracted from bone marrow or adipose tissue, while EPCs are generally isolated from peripheral blood or bone marrow. While these cells have the potential to be isolated from the targeted populations they may have altered function due to age or disease 39,40. Other cell types that are not involved in the natural bone healing process have also been investigated in bone tissue engineering, including embryonic stem cells 41 and induced pluripotent stem cells 42,43. Induced pluripotent stem cells are created from adult fibroblasts by the transduction of four genes that reprogram the cell back to a pluripotent stem cell phenotype 44. These cells can then be differentiated into bone and/or EC lineages for the formation of vascularized bone, which makes them of significant clinical interest.

Surgical practicality and cost-effectiveness are necessary for a technique to become standard of care. Strategies involving multiple surgeries or implant locations increase the risk of complications and associated costs. In addition, the medical community (surgeons, hospital administration, *etc*.) must be willing to adopt a new procedure. This may require that the treatment have significantly improved outcome in comparison to current standards of care. The decision is also influenced by trends, personal preferences, patient opinion and cost. Cost-effectiveness is an increasingly important consideration in any clinical treatment. Strategies that require extended *in vitro* culture, complex scaffold materials or preparation, or expensive proteins may place a significant financial burden on patients and/or the healthcare system. With continuously evolving healthcare systems, tissue engineers must proceed with careful consideration of approaches that may ultimately be cost-prohibitive.

In this review, we discuss literature on vascularized bone formation with an emphasis on these important clinical considerations. Various strategies based on cellular implantation, growth factor delivery, scaffold design and surgical pre-fabrication are described in more detail in the following sections. Each section includes a discussion of challenges to the clinical translation of the strategies.

## Cell-based strategies

Many approaches for engineering vascularized bone consist of a biomaterial scaffold seeded with cells. Cell types typically include an EC source (such as EPCs or HUVECs) and a bone source, often osteoblasts or stem cells (mesenchymal or adipose-derived). The scaffolds may be supplemented with soluble factors or matrix proteins in an attempt to further enhance tissue formation.

### Cells and scaffolds

Several groups have investigated the use of polymer scaffolds combined with a single cell source to engineer vascularized bone. There have been numerous studies in which MSCs (bone marrow or adipose derived) have been seeded on a scaffold, resulting in increased osteogenesis in model systems *in vivo* 45–48. These approaches do not directly attempt to build vessels within the scaffolds. Instead, they depend on host vessel ingrowth in response to the release of paracrine factors by the implanted MSCs. Mesenchymal stem cells, regardless of the source, release pro-angiogenic factors upon implantation, including VEGF, BMP-2, and Ang-1 12.

In these approaches, vascularization may also result from direct assembly of MSCs into vascular structures 49–51. Mesenchymal stem cells can participate in vessel assembly by functioning as perivascular support cells 49, or a subset of MSCs may be able to directly differentiate along the EC lineage 50,51. This phenomenon has been exploited to form vascularized structures using MSCs alone. Cell sheet constructs of bone marrow MSCs were shown to differentiate into both angiogenic and osteogenic lineages and form vascularized bone following implantation *in vivo* 52. Ossified trabeculae, woven bone and medullary cavities were all found in the newly developed bone 52. Endothelial cells derived from adipose derived stem cells (ASCs) have also been shown to improve vascularization of bone allografts in critical sized calvarial defects 53. In this case, it is not clear if the seeded cells directly form vascular networks or stimulate vascularization from surrounding host vessels *via* the release of angiogenic factors. Interestingly, combining these ECs with ASC-derived OBs in the same model resulted in less bone volume and microvessel density than ASC-derived ECs alone 53. This could result from the lack of sufficient porosity in the scaffolds potentially compromising the viability of the ASC-derived OBs 53.

A more common strategy is to seed cells from both osteogenic and endothelial lineages into a scaffold to coordinate new bone formation and vessel assembly. Demineralized bone matrix scaffolds seeded with both bone marrow-derived EPCs and MSCs resulted in a significantly higher blood supply, biomechanical strength and bone mineral density than scaffolds without EPCs when implanted in a segmental defect model 54. Mesenchymal stem cells combined with EPCs and seeded on polyurethane scaffolds with hydroxyapatite (HA) nanoparticles formed tubular structures *in vitro* after 7 days and exhibited earlier osteogenic differentiation than in monoculture 55. HUVECs formed elongated networks and stimulated increased early osteogenic differentiation of bone marrow MSCs on three-dimensional porous beta-tricalcium phosphate (β-TCP) scaffolds *in vitro* 56. Pericyte-like MSCs have been also shown to increase the stability of HUVEC networks within mineralized tissue 57.

Cell spheroids have been investigated in a variety of tissue engineering applications due to increased cell-cell interactions 58 and the ability to induce 3D sprouting of ECs 38. Osteoblast/human dermal microvascular endothelial cells co-culture spheroids implanted in a dorsal skinfold chamber model without any additional scaffold formed a vascular network that demonstrated initial inosculation by day 3 and extensive perfusion by day 14 59. HUVEC spheroids with osteoblasts seeded onto processed bovine cancellous bone (PBCB) scaffolds implanted subcutaneously in SCID mice formed dense, functional vascular networks that anastomosed with host vessels within the 21-day implantation period 60. In a following study, decalcified PBCB scaffolds seeded with HUVEC spheroids and osteogenically predifferentiated MSCs improved angiogenesis and promoted bone regeneration in a mouse cranial defect model 61.

These studies show that the combination of ECs and bone cells can increase both bone formation and vascularization. A more complex approach involves assembly of ECs into a vascular network in a scaffold *in vitro* prior to implantation. The goal of this approach is to achieve rapid and enhanced perfusion of the networks following implantation *in vivo* 62,63. Generation of a vascular network prior to initiating osteogenesis *in vitro* results in increased bone volume and vascular structures *in vivo* 64. HUVECs and MSCs were encapsulated in fibrin, seeded onto decellularized bone scaffolds, and then incubated for 2 weeks in endothelial growth media followed by 4 weeks with additional MSCs and osteogenic media 64.When these scaffolds were implanted, they exhibited bone formation with a vascular network that anastomosed *in vivo* in under 2 weeks 64. HUVECs seeded into collagen-glycosaminoglycan scaffolds formed networks within 6 days *in vitro* 65. The addition of MSCs to the HUVEC-seeded scaffolds halfway through *in vitro* culture resulted in enhanced vessel formation and higher vessel density following implantation *in vivo*, with the MSCs exhibiting a pericyte-like stabilizing role 65.

The periosteum is a rich source of vasculature and osteoprogenitor cells that is known to enhance bone formation and healing. A tissue engineered periosteum is an emerging strategy for enhancing the formation of vascularized bone. HA/poly(ester urethane) scaffolds surrounded by a platelet-rich plasma (PRP) gel and seeded with MSCs have been investigated as an engineered periosteal substitute 66. The engineered periosteum scaffold functioned as a source of growth factors and improved bone growth in rabbit ulnar defects after 4 months 66. This approach primarily treated the periosteum as a rich source of progenitor cells. However, the high vascular density of the periosteum is also important to its ability to enhance bone growth. Work in the area of engineered periosteum has not yet addressed the important issue of vascularity.

Despite the success of cell-based bone tissue engineering strategies in research settings, many of the techniques used model cell types (*e.g*. HUVECs) that are not a realistic cell source for clinical treatment of patients. In addition, the cells that are actually available in the potential patient population have substantial variability in osteogenic or angiogenic potential that could hinder translation into clinical application 28. For example, EPCs isolated from the peripheral blood exhibit increased angiogenic potential over those derived from the bone marrow 67. Age or co-morbidities may also affect the presence or function of cell sources in the patient population 68,69. Adipose-derived MSCs from aged patients with coronary artery disease exhibit decreased secretion of angiogenic factors 70. Additionally, prolonged *in vitro* culture expansion could decrease the proliferation, differentiation potential and bone formation potential of MSCs 71. These factors are significant questions that need to be answered for cell-based strategies.

### Cells and soluble factors

Cell-based therapies are often supplemented with growth factor delivery strategies designed to enhance cell function and integration. Growth factors commonly used in tissue engineering of vascularized bone include VEGF, BMP-2 and PDGF-BB. These are chosen for their beneficial effects on ECs, osteogenic cells or both 72. PDGF-BB secreted by ECs also plays a key role in recruitment and proliferation of vessel stabilizing pericytes 73. These soluble factors can be introduced to a system through gene therapy and/or polymer delivery systems.

A number of combined cell and growth factor delivery systems have been investigated for tissue engineering of vascularized bone *in vitro* and *in vivo*. Addition of physiologically relevant concentrations of PDGF-BB to growth media enhanced both angiogenesis and osteogenesis *in vitro* in ASC spheroids encapsulated in fibrin gels 74. Degradable poly(DL-lactic acid) scaffolds encapsulated with VEGF and seeded with bone marrow MSCs exhibited increased bone volume and blood vessel formation following implantation *in vivo* 75. In lieu of adding a single growth factor, multiple soluble factors can be delivered simultaneously through the use of PRP 76. Platelet-rich plasma consists of plasma and platelets from autologous blood and contains soluble factors secreted by platelets, including PDGF, VEGF, IGF-1 and von Willebrand Factor 76. Platelet-rich plasma-loaded alginate microspheres seeded with ASCs exhibited enhanced mineralization and formation of an anastomosed capillary network *in vivo* 77.

Gene therapy is often used to target a sustained delivery of growth factors 78. Mesenchymal stem cells transduced with a recombinant adenoviral vector carrying BMP-2 displayed increased ALP activity, type I collagen expression, matrix mineralization and bone formation *in vitro* and *in vivo* 79. Hypoxia inducible factor-1 (HIF-1α) regulates oxygen homeostasis, targets VEGF and activates the transcription of several angiogenic genes 80,81. Mesenchymal stem cells overexpressing HIF-1α and seeded within gelatin sponge scaffolds exhibited significantly upregulated expression of angiogenic factors *in vitro* and created substantial blood vessel networks within mineralized tissue *in vivo* 81. Bone marrow MSCs genetically modified to over-express VEGF seeded in a scaffold of silicate-substituted apatite granules in a fibrin gel created dense vascular networks in nude rats, though this resulted in a reduced quality of bone mineralization 82. This may result from increased degradation of bone due to increased osteoclast differentiation 82. Other studies with VEGF-overexpressing cells did not report this phenomenon 83, indicating that more research into the mechanisms behind this shift in bone homeostasis is necessary.

Though these techniques show potential for clinical success, cost and safety remain significant considerations that may inhibit clinical implementation. Applications of large amounts of soluble factors and cells even for relatively small defects may be costly. Cells that overexpress soluble factors may be an efficient method for growth factor delivery, but the paracrine effects of high growth factor concentration could be a concern. Ectopic growth in untargeted regions is also a risk of growth factor therapies as has been seen clinically for studies involving BMPs 84.

### Bioreactors

Bioreactors have been widely investigated in tissue engineering to enable long-term culture of large engineered tissues. The techniques are typically focused on enhancing nutrient transport in scaffolds in the absence of functional vascular networks. Several weeks of culture is often necessary for optimal tissue growth and development. For bone applications, bioreactors may also enhance osteogenic differentiation of MSCs due to shear stress resulting from media flow 85.

Cell behaviour in the bioreactors depends on a variety of factors, including flow conditions, cells used, and biomaterial environment. Steady flow followed by pulsatile flow increased mineralization and mechanical strength of ASC-seeded porous silk fibroin scaffolds in a bioreactor 86. A tubular perfusion bioreactor was shown to enhance osteogenic differentiation and mineralization of MSCs encapsulated in alginate beads as a function of flow rate 87. Perfusion culture resulted in a more uniform distribution of cells and matrix in comparison to static conditions when HUVECs and MSCs (in monoculture or co-culture) were seeded onto electrospun poly(ε-caprolactone) (PCL) scaffolds and incubated in osteogenic medium 88. In addition, increased mineralization was observed in perfusion MSC cultures relative to static co-culture 88. This was not seen in perfusion co-culture, possibly due to shear stress affecting the function of HUVECs 88, as EC function varies with shear stress 89.

While the strategies discussed above have shown that bioreactors can be used to enhance engineered bone formation, there has been little investigation into formation of vascular networks within scaffolds in a bioreactor setting. In one study, MSCs and MSC-derived ECs seeded on a porous poly(lactic acid) scaffold assembled into vascular-like structures within bone tissue in a rotating wall vessel bioreactor 90. Endothelial cells were grown in the bioreactor in EGM for 1 week prior to the addition of MSCs and the induction of osteogenic differentiation for an additional week 90. Additionally, the co-culture bioreactor conditions exhibited increased spatial distribution and proliferation relative to static controls 90.

Bioreactor strategies have seen some success in clinical studies, but these efforts have not yet transitioned to clinical use for bone tissue engineering. A primary concern is cost. The cost of a clinical bioreactor bone graft has been estimated to be $10,000–$15,000, taking into account the cost of cells, labour, testing, miscellaneous expenses and a portion of the initial cost to set up a bioreactor system (estimated at $25,000–$35,000) 91. However, this is only an estimate of the graft cost and does not include surgical and hospital costs that would also be incurred for the procedure. In addition, the large volumes of tissues potentially generated in a bioreactor will need to be combined with strategies that promote rapid vascularization in order for the constructs to survive post-transplantation. Bioreactor strategies will likely need to implement approaches for creating vascular networks within the scaffolds for successful clinical implementation. However, the high costs and long culture times present a significant challenge to ultimate clinical acceptance.

## Cell-free strategies

Not all tissue engineering strategies focus on the application of isolated cells. Cell-free strategies avoid issues of cell sourcing by focusing on the ability to induce surrounding cells to invade and generate bone of sufficient volume in time frames appropriate for clinical success. Typically, this is done through the delivery of growth factors or the application of specially designed bio-active scaffolds.

### Growth factor delivery

VEGF is one of the most widely investigated growth factors for controlled stimulation of angiogenesis. An injectable alginate hydrogel releasing VEGF was shown to enhance angiogenesis in a rodent cranial defect model and concomitantly enhanced bone regeneration in the absence of additional cells 92. Phosphonic acid self-assembled monolayers (SAMs) were used to modify HA scaffold surfaces to bind VEGF to the interior surface of the scaffold as another sustained delivery strategy 93. Human aortic ECs seeded onto the VEGF-bound, SAM-coated scaffolds saw an increase of proliferation and angiogenic activity compared to HA scaffolds alone, with VEGF remaining on the surface for up to 28 days *in vitro* 93.

**Figure 2:**
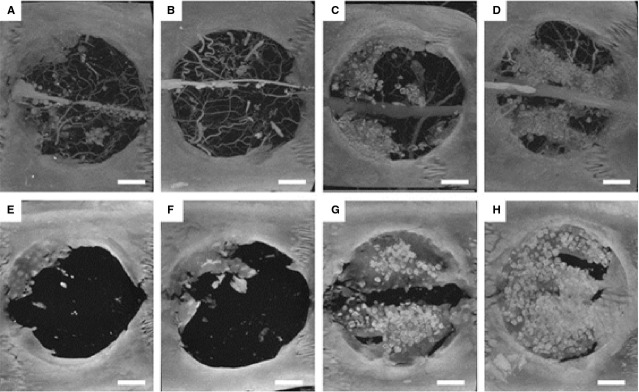
MicroCT images of cranial defect sites at 4 weeks (A–D) and 12 weeks (E–H) display evidence of bone and blood vessel formation. Microfil perfusion was performed to visualize blood vessels for 4 week samples but not 12 week samples. Groups include blank (A and E), VEGF only (B and F), BMP-2 only (C and G) and dual VEGF/BMP-2 (D and H). Scale bar represents 200 μm for all panels. Figure reproduced with permission, from Patel *et al*. 95.

Bone morphogenetic protein-2 has been studied extensively in bone tissue engineering due its significant role in the induction of bone formation 21. It is also known to possess pro-angiogenic properties 19 and play a role in the crosstalk between EPCs and MSCs 22. Critical-sized femoral segmental defects in rats were treated with an injectable alginate hydrogel for BMP-2 delivery combined with an electrospun PCL nanofibre mesh for guided bone regeneration 94. Bone healing was observed after 4 weeks and substantial bone formation after 12 weeks 94. VEGF and BMP-2 can also be delivered simultaneously to stimulate bone and vascular network formation. Dual-delivery of VEGF and BMP-2 *via* gelatin microparticles within a porous poly(propylene fumarate) scaffold resulted in increased bone and blood vessel volume in a rat cranial defect model (Fig.2) 95. Though VEGF and BMP-2 dual delivery had similar amounts of bone formation as BMP-2 alone, dual delivery may enhance bone bridging and union of the defect 95. This synergistic effect may be model-specific. In a study using lower doses of VEGF and BMP-2, the effect of BMP-2 on bone growth was found to be dose-dependent 96. The addition of higher amounts of VEGF did not offset the decreased bone formation observed with low concentrations of BMP-2 96. The effects of BMP-2 and VEGF dual delivery may also be dependent on location and rate of release 97.

Growth factor delivery treatments appear successful in many small volume pre-clinical studies, but large volume bone defects present additional challenges that must be overcome. Many strategies have only been tested in small volume applications, and scaling up for large volume defects may present issues. Vascular in growth from the host may be too slow to overcome ischaemia throughout the entire implant volume. Treatments with multiple growth factors may benefit from synergistic interactions, however, the cost of recombinant proteins is high. The requirement of greater amounts of proteins for large volume defects will only further increase price. Researchers need to identify the minimal elements needed for success and may be able to reduce dose through the use of controlled delivery strategies.

### Scaffold design

Optimizing the bioactivity of scaffolds to encourage bone and/or vessel formation is another critical component of bone tissue engineering. Scaffold design techniques include developing new materials, investigating novel fabrication methods or optimizing mechanical or physical properties to improve osseointegration and vascularization 98.

Bioactive glass 99 has been utilized in bone tissue engineering applications due to its osteoconductive properties. Additionally, bioactive glass has also been shown to have pro-angiogenic properties, particularly 45S5 glass (a silicate-based glass) 100. Direct or indirect cellular contact with 45S5 glass can result in increases in angiogenic indicators 100, which makes this material of particular interest for vascularized bone applications. Bioactive glass scaffolds of various compositions were implanted in a rat cranial defect model for 12 weeks and displayed new bone formation, HA conversion and blood vessel infiltration 101. Samples with 45S5 glass had highest blood vessel area, while 1393B3 (borate-based) had the highest amount of bone formation and converted completely to HA 101. Bioactive glass foam scaffolds of 70S30C (70% SiO_2_, 30% CaO) composition demonstrated evidence of remodelling by osteoclasts as well as supported EC tube formation *in vitro* 102. Mesoporous bioactive glass scaffolds may offer increased bioactivity and degradation 103, and can also be used for drug delivery. One study combined osteogenic mesoporous bioactive glass with a strategy to induce angiogenesis through the induction of HIF-1α *via* cobalt ions 104. Hypoxia-mimicking mesoporous bioactive glass scaffolds were created by incorporating 2% or 5% Co^2+^ ions to replace parts of Ca^2+^ ions, then seeded with bone marrow stromal cells and cultured for 7 days *in vitro* 104. Cells extracted from these scaffolds exhibited significantly increased HIF-1α and VEGF gene expression and VEGF secretion, suggesting the induction of the hypoxic cascade, which may stimulate neovascularization *in vivo* 104.

Scaffolds have been designed with physical and chemical features designed to enhance bone formation. The degradation rate of biomaterials can influence tissue development. Hyaluronic acid hydrogels with controlled degradation properties have been investigated to encourage oriented bone growth when combined with soluble factors 105. Hydrogels loaded with BMP-2 and/or VEGF were implanted into a rat cranial defect model for up to 6 weeks 105. Fast-degrading BMP-2 loaded hydrogels had increased oriented collagen area compared to slow and intermediate degradation rate 105. Dual delivery of BMP-2 and VEGF in fast-degrading hyaluronic acid hydrogels resulted in increased mineral volume over BMP-2 and VEGF alone 105. Shell-core bi-layered PCL scaffolds developed to mimic osteon structure were able to stimulate bone tissue formation in the shell and blood vessel formation in the core region 106. Seeding the inner core with mouse ECs and the outer shell layer with mouse pre-osteoblasts resulted in osteogenic differentiation of the pre-osteoblasts and formation of a continuous lining of ECs mimicking Haversian canals 106. 3D-printing allows for the rapid generation of custom-shaped scaffolds from a variety of starting materials. 3D-printed porous PCL scaffolds seeded with ASC aggregates suspended in fibrin gel formed integrated vascularized tissue with dense mineral deposits within the scaffolds after 2 weeks of culture *in vitro* 107. Scaffolds were designed with uniform pore size and fibre width with 40% infill density to support cellular infiltration and allow for uniform distribution of cellular aggregates throughout the pores 107. After subcutaneous implantation for 1 week *in vivo*, scaffolds seeded with ASC aggregates exhibited increased cellularity and vascular density, particularly within the centre of the scaffold 107. Vessel formation was further increased in scaffolds that were pre-vascularized for 18-days *in vitro* prior to implantation 107. These scaffolds can also be created in the shape and volume of a human mandible and maxilla from computerized tomography (CT) scans 107, indicating the potential for patient-specific scaffolds to be used clinically.

Using bioactive materials to induce bone formation may be an effective treatment method that avoids the cost and risks associated with cells and soluble factors. However, many of these strategies may need to be supplemented with cells or soluble factors to optimize bone volume. Designing scaffolds to best mimic the structure of bone and its vasculature shows potential to speed bone formation by increasing the availability of nutrients and progenitor cells. The recent widespread popularity of 3D printing may make these techniques more accessible and clinically feasible, and could lead to further improvements in the design of patient-specific scaffolds.

## Surgical approaches

Tissue engineering has often progressed with the goal of engineering ready-to-implant, fully functional tissues. These strategies sometimes neglect the inevitable remodelling process that occurs following implantation 108. Surgeons, on the other hand, have a long history of utilizing the body's own healing and inflammatory processes to enhance tissue vascularization. Recently, surgical approaches have been used to assist in enhancing construct vascularization within the patient prior to implantation at the defect site 109. These techniques have the advantages of exploiting the patient's own healing capacity by implantation in an ectopic location selected, in part, on an ability to enhance vascularization.

A vascular bundle inserted within a scaffold can help prefabricate the construct to improve vascularization and bone formation. An *in vivo* bioreactor was created in rabbits by implanting a tissue engineering strategy around the saphenous vessel bundle and wrapping it with the *muscularis* membrane 110. Application of β-TCP granules embedded with BMP-2 modified bone marrow MSCs in this model resulted in active bone formation with an increased capillary density made from autologous cells after 4 weeks 110. β-TCP scaffolds created with a groove to house the femoral vascular bundle were seeded with osteogenically differentiated MSCs 111. The MSCs were differentiated for 3 weeks *in vitro* and allowed to adhere on scaffolds overnight prior to implantation in critical-sized segmental femoral defects in rabbits 111. Bone remodelling with a bone marrow cavity was observed after 8 weeks. Prefabricated scaffolds had higher vascular density with more spatial uniformity, whereas scaffolds without prefabrication had vessels localized primarily at the periphery of the scaffold 111.

A surgically induced periosteal membrane takes advantage of the body's natural healing processes and has been characterized in humans 112. To induce membrane growth, a poly(methyl methacrylate) (PMMA) cement spacer is placed within a critical sized bone defect and removed 6–8 weeks later 112,113. A membrane grows around this spacer, and, similar to native periosteum, it contains MSCs, ECs, and growth factors essential for bone regeneration 112. The induced periosteal membrane has been shown to prevent resorption of implanted cancellous bone graft and encourage vascularization 113. While this has not been explored previously, a tissue engineering strategy could also be implanted into this optimized healing environment. A one-step procedure could be possible if a tissue engineering strategy was able to induce membrane growth in place of the PMMA spacer. An engineered periosteum may further accelerate the healing process by emulating the role of native periosteum in autograft healing 114. Bone allografts were coated with degradable poly(ethylene glycol) hydrogels in a murine segmental femoral graft model to deliver MSCs and act as a mock periosteum 115. Defects treated with tissue engineered periosteum exhibited increased vascular volume, bone callus formation and mechanical stability compared to untreated allografts 115.

**Figure 3:**
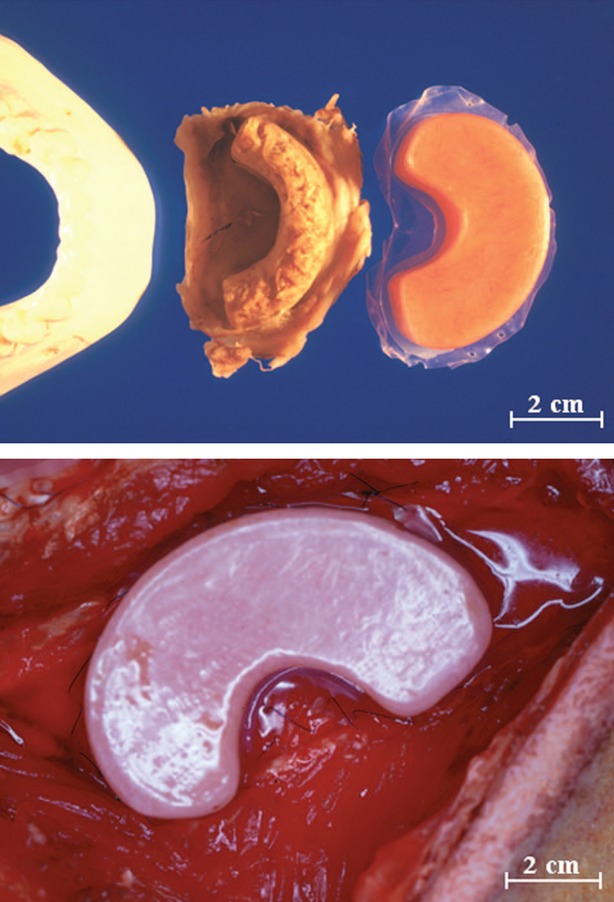
(Top) Poly(methyl methacrylate) chambers designed to mimic the size and shape of the mental protuberance of the mandible. (Below) Chambers filled with autologous morselized bone graft were implanted in sheep rib with the open side exposed to the cambium surface of the periosteum. Scale bar represents 2 cm. Figure reproduced with permission, from Cheng *et al*. 117.

Existing periosteum can be exploited as a source of osteoprogenitor cells and vasculature to enhance ectopic bone formation. Following ectopic growth and development of a tissue engineering chamber implanted against the periosteum, the new tissue can then be transferred to the defect site as a vascularized tissue. Chambers containing autologous morselized bone graft (MBG) implanted against rib periosteum in sheep generated significant calcified tissue within the chambers, with maximal after 8 weeks of implantation 116. A similar approach was used to generate the mental protuberance of the mandible, using anatomically shaped chambers (Fig.3) 117. Active bone formation and calcified tissue area increased over 12 weeks of implantation, with chamber volume peaking at 9 weeks prior to significant tissue regression 117. This procedure was translated clinically in a patient to augment mandible height during reconstruction 118. An MBG-filled chamber was implanted against rib periosteum for 8 weeks, at which time the bone graft and periosteum were harvested and transferred to the mandible. The engineered bone graft remained viable after 16 months, and histology showed the formation of compact bone with numerous Haversian systems and mature osteocytes 118.

One approach that has been studied extensively for other engineered tissues is the arteriovenous-loop model 119. In this procedure, the saphenous artery and vein are microsurgically dissected and anastomosed together to form a loop, which is placed inside a custom-made isolation chamber containing a tissue engineering strategy and fixed to the underlying fascia 120. The AV-loop provides large vessels within the engineered tissues, enhances vascularization, and can be used later for microsurgical tissue transfer to a defect location. β-TCP-HA granules with MSCs and recombinant human BMP-2 were implanted in sheep around an arteriovenous-loop for 12 weeks 121. Mature bone formation was observed with evidence of active remodelling, along with a dense vascular network 121, demonstrating the potential for this model to be used for creation of vascularized transplantable bone.

Space maintainers can be used in conjunction with these strategies to preserve and enhance a defect site while the bone graft is grown at an ectopic location 122,123. In a rabbit composite mandibular defect model, porous PMMA space maintainers inserted at the defect site exhibited enhanced soft tissue healing and implant coverage over solid implants 122. The space maintainer preserves the soft tissue envelope surrounding the defect, acts as a template for soft tissue regrowth, and prevents scarring at the defect site 123,124. These implants could also be coupled with antibiotics or soluble factors to further improve defect healing and minimize risk of infection 123.

Surgical solutions for vascularized bone tissue engineering may help to enable the translation of basic research strategies into the generation of large bone volumes. However, these techniques have not been exploited significantly in the field of tissue engineering. In some cases, these techniques utilize autologous cells and proteins without extensive *in vitro* culture. Two-step surgical procedures may be the required strategies for further translational clinical applications, however, these strategies introduce additional risks and hospitalization costs.

## Conclusion

Many recent advances have been made towards engineering vascularized bone. The widespread utilization of autologous, clinically available cells is encouraging for the clinical translation of these methods. Additional insight into the complex interactions between osteogenic and ECs may lead to future success in cell-based strategies. Another major hurdle in developing tissue engineered vascularized bone grafts is scaling up to the appropriate volume. Many of the strategies discussed here result in small volumes of tissue on the order of millimetres, whereas defects are often on the centimetre scale. Surgical strategies are able to create larger volumes, but often require multiple surgeries or surgical sites, increasing risk of infection and other complications. Bioreactor-based approaches are designed to result in larger volumes, but high costs and long culture times could hinder their clinical implementation. The ultimate clinical success of tissue engineered vascularized bone requires novel strategies to overcome these challenges.
